# Moderating effects of demographic characteristics on the relationship between parenting practices and energy balance related behaviors of Chinese preschoolers

**DOI:** 10.3389/fpubh.2024.1476733

**Published:** 2024-12-24

**Authors:** Zhou Peng, Patrick Wing-chung Lau, Li Ming Wen

**Affiliations:** ^1^Department of Sport Physical Education and Health, Hong Kong Baptist University, Kowloon, Hong Kong SAR, China; ^2^School of Public Health, The University of Sydney, Sydney, NSW, Australia

**Keywords:** physical activity, diet, sleep, screen time, child-rearing behaviors, preschool-age children

## Abstract

**Background:**

Early establishment of energy balance related behaviors (EBRBs) may be effective in combating unhealthy lifestyle in preschoolers. Parents are responsible for cultivating preschoolers’ EBRBs directly through parenting practices. Although investigating the impact of various parenting practices on preschoolers’ EBRBs is crucial to determine which practices should be recommended to parents to help reverse childhood unhealthy lifestyle, it is important to assess whether these effects of parenting practices on preschoolers’ EBRBs would be similar across different groups of preschoolers, necessitating research into the moderating effects of demographic characteristics.

**Methods:**

Baseline dataset was utilized from an electronic health intervention study of Chinese 3-6-year-old preschoolers. Preschoolers’ PA and sleep duration and quality were objectively examined using wGT3X ActiGraph accelerometer. Data related to parents and preschoolers’ eating behaviors and sleep problems were subjectively assessed using parent-reported questionnaires. The PROCESS macro version 4.2 was used to perform moderation analysis. When the interaction revealed a *p*-value of less than 0.05 and the 95% confidence interval did not include 0, the conditional effects of the focal predictor at various levels of the moderator were further examined (*p* < 0.05) to investigate how the relationship between parenting practices and preschoolers’ EBRBs varied with different moderator levels.

**Results:**

Married parents exhibited the desired outcomes in the relationship between parenting practices and their preschoolers’ EBRBs. In contrast, divorced/separated parents showed unfavorable results in this relationship. The association between the parents’ and preschoolers’ BMI varied depending on parents’ socioeconomic status, the preschoolers’ age, and the number of children in the household. The preschoolers’ age showed different moderating trend on the relationship between parents’ PA and preschoolers’ sedentary behaviors as well as the association of eating-related PSE with preschoolers’ eating behaviors. The mechanisms linking the parents’ PSE to the preschoolers’ PA, sedentary behaviors, and sleep duration were influenced by the preschoolers’ gender and the number of children in the household.

**Conclusion:**

The potential parental influence warrants further investigation with the consideration that the relationship between parenting practices and preschoolers’ EBRBs varied across different group of children.

**Clinical trial registration:**

NCT06025019.

## Introduction

Preschoolers’ lifestyles have become increasingly characterized by physical inactivity, sedentary behaviors, unhealthy eating habits, disrupted sleep routines, and excessive screen time (1–5). Unhealthy lifestyle behaviors formed in the early childhood can track throughout the lifespan and may even contribute to the obesity and non-communicable diseases (e.g., cardiovascular diseases, diabetes, and chronic lung diseases) (6–8). Energy balance related behaviors (EBRBs) consist of physical activity (PA), dietary behaviors (DB), sleep and screen time (ST) (9). The early cultivation of health EBRBs, alongside with their synergistical effect, could be effective in reversing the unhealthy trend of lifestyle in the early childhood (e.g., adherence to recommended guidelines for PA, eating, sleep, and ST) (10). Parental involvement has played an important role in forming preschoolers’ EBRBs because parents may affect preschoolers’ EBRBs directly through parenting practices, which are identified as the practices parents use to effectively influence their children’s behaviors such as support and engagement in PA, role modeling in healthy eating, establishing the regulations of sleep and ST (11), while preschoolers are still developing their autonomy and rely on parental supervision (12).

Extensive studies have examined the relationship between parenting practices and preschoolers’ EBRBs. For example, previous study conducted in Canada found that parental support, encouragement to be physically active, and monitoring PA were associated with increased preschoolers’ PA (13). Restricting intake of unhealthy food was related to higher intake of those food and higher Body Mass Index (BMI) in the US and Sweden (14, 15). Philbrook (12) who explored a pathway linking the parental involvement at bedtime and preschoolers’ nighttime sleep duration and quality in the US found that longer parental presence and contact at bedtime were associated with better sleep (i.e., longer sleep time and greater sleep efficiency). Another study from the US indicated that restriction of child’s ST has found to be related to decrease weekly TV watching (16). Specific parenting behaviors arise from both cultural influences and the stimuli associated with the process of acculturation. Varying social values can lead to different socialization strategies aimed at helping children align with cultural norms. It can be inferred that parents with varying cultural values may adopt different parental values and practices (17). Consequently, the parental correlates of preschoolers’ PA, diet, sleep, and ST in western countries could not be generalized to other cultural contexts. Besides, given that EBRBs are not isolated but exhibit a tendency to co-occur in the early childhood (10), a clustered analysis of their parental influences is warranted, rather than examining them individually. In this perspective, comprehensive investigations on the relationship between parenting practices and preschoolers’ EBRBs within specific cultural context such as China are crucial to determine which practices should be recommended to parents to help combat unhealthy lifestyles of Chinese preschoolers.

The theoretical model proposed by Darling and Steinberg (18) argues that the influence of parenting practices on children’s development depends on children’s personality traits and parental factors. With regards to children’s traits, parents’ food restriction has been found to have undesirable impact on dietary intake of girls than of boys (14). Controlling diet-related parenting practices was positively related to favorable dietary consumption for normal weight children, but not for the overweight children (19). Also, the relationship between restrictive feeding practices and desirable dietary intake in preschoolers was found to be weaker or even non-existent among children with problematic eating styles, such as those who are picky eaters, reluctant to eat, or slow eaters (19). Regarding the parental factors, pressure to eat was found to be positively related to children’s dietary intake when parents were mother (20), had lower socioeconomic status (21), were older age (22), were higher BMI (23), and had higher educational level (24). Empirical evidence regarding the moderators of parenting practices on preschoolers’ EBRBs is generally lacking in Chinese cultural contexts, an examination of the interactions of parents and child characteristics with parenting practices may further help identify which parents use what practices can effectively improve preschoolers’ EBRBs, potentially informing the design of family-based programs and policies aimed at promoting healthy EBRBs in preschool-aged children.

The current study aims to explore whether the relationship between parenting practices and preschoolers’ EBRBs would be moderated by demographic characteristics, which in the present study refers to preschoolers’ age and gender as well as parents’ marital status, income, number of children in the household, and educational level. The hypothesis is that these children’s and parents’ demographic characteristics would moderate the association of corresponding parenting practices with preschoolers’ EBRBs.

## Methods

### Overview

The current study utilized baseline dataset (*n* = 206) from an electronic health intervention study of Chinese preschoolers aged from 3 to 6 years, which was designed as a two-arm single-blinded randomized controlled trial. *A priori* analysis was conducted using G*power 3.1.9.6 (25), a minimum sample size of 82 parent–child dyads in intervention and control group, respectively, is required to detect a small effect size d of 0.3 with a power of 0.80 under the condition of an alpha level of 0.05. Considering the 20% potential attrition rate, 206 parent–child dyads in total are needed (103 parent–child dyads in each group). The protocol for this parent-based eHealth intervention study has been published elsewhere (26). Data was collected from October 2023 to June 2024 and consisted of baseline, posttest, and follow-up, with a 12-week interval between each assessment. Parent–child dyads were recruited from four kindergartens in Guiyang, Guizhou province, China, with the assistance of Sun Yat-sen University and Guiyang Preschooler Education College. The inclusion criteria were [1] parents should be over 21 years old with children between the ages of 3 and 6; [2] parents and children should be in a healthy condition based on the health assessment conducted by kindergartens at the beginning of the new semester (referring to a state of physical, mental, social, intellectual, and emotional well-being and the absence of disease); [3] parents and children should reside together for at least 4 days per week. One face-to-face meeting was arranged in each of kindergartens to provide more details of this study. Parents/guardians were required to sign an informed consent form at the end of the parent meetings.

#### Measurements

##### Demographic information

**Parents**: socioeconomic status, age, gender, marital status, number of children in the household and Body Mass Index (BMI) were collected. Parent’s BMI was calculated based on the self-reported values of their weight and height (height/weight^2^), the results were then categorized using cutoff points for adults (i.e., 18.5 < BMI <23 indicating normal weight, BMI ≥ 23 indicating overweight or obesity) (27).

**Preschoolers**: child’s sex, age, weight, height, and BMI. Preschoolers’ weight was measured using standard practices to the nearest 0.1 kilograms using calibrated measurement scales (Salter) and height was measured using a stadiometer (SECA) to the nearest 0.1 cm. BMI was calculated with height and weight measurements (height/weight^2^). The BMI score was then categorized into overweight or obesity based on age and gender standard provided by the International Cutoff Points for Identification of Overweight and Obesity in Children (kg/m^2^) (28).

##### Preschoolers’ physical activity and sedentary behaviors

The PA level was objectively measured using a tri-axial accelerometer ActiGraph GT3X-BT, which has been validated and proven reliable for assessing PA levels in preschoolers (29). Both kindergarten teachers and parents received written and video instructions on how to use the accelerometer. Parents were also instructed to keep an activity diary to record both wear and non-wear time of the accelerometer. Teachers checked the accelerometer wear on each school day. The accelerometer was worn on the participant’s right wrist continuously for 7 days, except during water-related activities. Data was downloaded and processed using the ActiLife software (version 6.13) and analyzed in 1-s epochs. Valid wear time was defined as a minimum of 16 h of wear time over at least 3 days, including two weekdays and one weekend day (30). Non-wear time was determined as 60 consecutive minutes with zero counts per minute, following standard approaches in ActiLife. The accelerometer’s activity counts were categorized into different intensities by using the cut-off points: sedentary: <819 counts per minute (CPM); light: 820–3,907 CPM; moderate: 3908–6,111 CPM; and vigorous: ≥ 6,112 CPM.

##### Preschoolers’ dietary behavior

Children’s dietary behaviors was evaluated using the Children’s Eating Behavior Questionnaire, which has been validated in Chinese preschool-age children (31). Parents rated the frequency of their child’s eating behaviors on a 5-point scale (1 = never, 2 = rarely, 3 = sometimes, 4 = often, 5 = always) across eight domains comprising 35 items. These domains include satiety responsiveness (e.g., My child gets full before his/her meal is finished), slowness in eating (e.g., My child finishes meal quickly), food fussiness (e.g., My child enjoys tasting new foods), food responsiveness (e.g., if allowed to, my child would eat too much), enjoyment of food (e.g., My child enjoys eating), desire to drink (e.g., if given the change, my child would always be having a drink), emotional undereating (e.g., My child eats more when he/she is happy), and emotional overeating (e.g., My child eats more when she/he has nothing else to do). The scales demonstrate high internal consistency reliability, with an overall Cronbach’s alpha above 0.7.

##### Preschoolers’ sleep duration and quality

The ActiGraph was utilized to investigate the duration and quality of children’s sleep, in combination with a log sheet completed by parents and a nap schedule provided by kindergartens. Bedtime and wakeup time were determined using an algorithm developed by Sadeh et al. (32) and periods of sleep were estimated using algorithm developed by Tudor-Locke et al. (33). The ActiLife software (version 6.13) was employed to assess the duration of nighttime sleep, daytime naps, bedtime and wakeup time, sleep efficiency, and sleep latency using 60-s epochs. These data were then matched to the nap schedule recorded by kindergarten teachers and the log sheets completed by parents. The total daily sleep duration was calculated by summing up both nighttime and daytime sleep periods.

##### Preschoolers’ sleep problems

Chinese version of the Children’s Sleep Habits Questionnaire (34) was employed to evaluate sleep problems among preschoolers. It consists of 33 items that assess eight different domains related to sleep, including bedtime resistance, sleep onset delay, sleep duration, sleep anxiety, night waking, parasomnia, sleep-disordered breathing, and daytime sleepiness. Each item is scored on a 3-point scale: “usually,” “sometimes”and “rarely’. Scores higher than 41 indicate a sleep problems. The CSHQ has demonstrated good reliability (Cronbach’s Alpha is 0.73) and validity.

##### Preschoolers’ screen time

Parents were asked to estimate the usual amount of screen time for their children on a typical weekday and weekend to determine the average screen time per week. For example, during the usual week, how much time does your child spend watching TV at home on weekdays and weekends? Questions were also involved the availability of screens and rules about screen entertainment. This questionnaire was used in previous study (35).

##### Parent’s physical activity

The Chinese Version of the International Physical Activity-Short Form was utilized to assess the PA levels of parents. This questionnaire consisted of seven questions that inquired about the amount of time spent engaging in PA over the past 7 days. The responses were then converted into metabolic equivalent task (MET) scores for each dimension or intensity of PA. To calculate the weekly PA in MET-minutes/week, the total number of minutes spent in each activity category was multiplied by the specific MET score assigned to that activity. Walking was assigned a MET score of 3.3, moderate physical activity (MPA) was assigned a score of 4 METs, vigorous activity (VPA) was assigned 8 METs. The questionnaire has demonstrated good reliability and validity in China, with an overall Cronbach’s Alpha of 0.79 (36).

##### Parenting feeding style

The Chinese Version of the Parent Feeding Style Questionnaire was utilized to assess parenting feeding styles. The questionnaire consists of four parts: instrumental feeding (IF; 4 items), emotional feeding (EF; 5 items), prompting or encouragement to eat (EnF; 8 items), and control over eating (CE; 10 items). Respondents were asked to rate their agreement on a 5-point Likert scale, ranging from “never” to “always.” For instance, “I allow my child to choose which food to have for meals.” The average score for each scale was calculated, with higher scores indicating a greater inclination for parents to engage in a particular feeding style. The overall Cronbach’s Alpha reliability coefficient for the questionnaire is 0.75 (37).

##### Parents’ self-efficacy

Parents’ self-efficacy (PSE), identified as parent’s belief in their ability to successfully perform the parenting role in their child’s PA, DB, sleep, and ST (38), was evaluated using a questionnaire previously used by Hammersley et al. (39). The questionnaire was back-to-back translated into simplified Chinese. It consists of 13 questions, with 4 items related to PA, 6 items related to DB, 1 item related to sleep, and 2 items related to ST. For example, one question asks how confident parents are in promoting healthy eating habits for their child. Respondents were asked to rate their confidence level on a 10-point Likert scale, ranging from “not at all” to “to a very high degree.” The sum of the scores for the 13 questions reflects the overall self-efficacy of parents in managing their child’s PA, DB, sleep, and screen time. A higher total score indicates greater self-efficacy in these areas.

##### Statistical analysis

All analyses were conducted using IBM SPSS (version 29), *p* value <0.05 was considered statistically significant. Number and percentage were performed on all demographic variables. Mean, median, standard deviation, number and percentage were performed on all outcome variables. PROCESS Marco version 4.2 by Andrew F. Hayes was utilized to conduct moderation analysis. The parenting practices were employed as independent variables while preschoolers’ EBRBs were set as dependent variables, the demographic characteristics were potentially set as moderators, which include preschoolers’ age and gender as well as parents’ marital status, income, number of children in the household, and educational level in the current study. When the interaction indicated *p* value <0.05 and 95 confidence interval did not include 0, the conditional effects of the focal predictor at values of the moderator were further checked (*p* < 0.05) to explore the relationship between parenting practices and preschoolers’ EBRBs depending on the different level of moderators, which were visualized using Microsoft Excel version 16.

## Results

**Figure 1:**
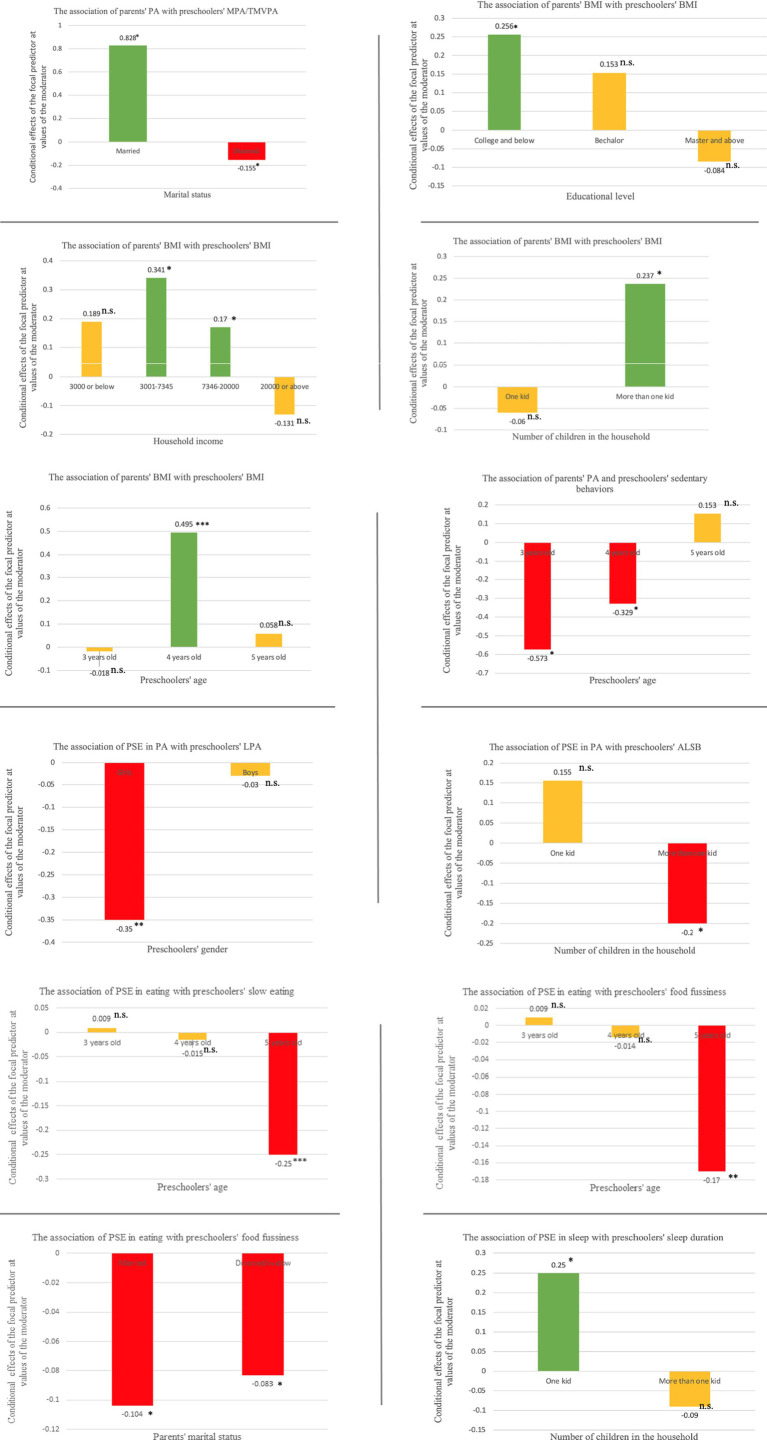
Significant demographic characteristics moderating the association between parenting practices and preschoolers’ EBRBs (*p < 0.05; **p < 0.01; *p* < 0.001. n.s, not significant). *The green bars represent the positive associations between parenting practices and preschoolers’ EBRBs, while the red bars indicate the negative association between parenting practices and preschoolers’ EBRBs. Yellow bars represent the insignificant relationship under this condition. *TMVPA, total moderate to vigorous physical activity; LPA, light physical activity; ALSB, average length of sedentary behaviors. *Because none of preschoolers engaged in vigorous physical activity at baseline, thus resulting in the same values of MPA and TMVPA.

A total of 327 parents attended the four face-to-face meetings. Of which, 237 parents signed the informed consent. All the participating parents completed questionnaires collection and 196 participating preschoolers provided valid ActiGraph data. An overview of demographic characteristics of preschoolers and their parents and outcome variables can be found in Table 1. Figure 1 shows how the significant relationship between parenting practices and preschoolers’ EBRBs varies across the different values of moderators.

**Table 1:** Demographic information and outcome variables of participating parent–child dyads.

	Characteristics	Category	Number (%)
Preschoolers demographic information	Gender	Girl	101 (42.6)
		Boy	136 (57.4)
	Age	3	31 (13.1)
		4	78 (32.9)
		5	128 (54)
	BMI	Under/normal weight	191 (80.6)
		Overweight and obesity	46 (19.4)
	Number of children in the household	1	73 (30.8)
		2 or more	164 (69.2)
Parents demographic information	Age	≤30	36 (15.2)
		31–35	120 (50.6)
		36–40	59 (24.9)
		≥41	22 (9.3)
	Gender	Mother	170 (71.7)
		Father	66 (27.8)
		Grandparent	1 (0.4)
	BMI	Under/normal weight	188 (79.3)
		Overweight or obesity	49 (20.7)
	Education level	Primary	16 (6.8)
		Junior high school	3 (1.3)
		Senior high school	30 (12.7)
		College	54 (22.8)
		Bachelor	112 (47.3)
		Master or above	22 (9.3)
	Household income (RMB/per month)	≤3,000	15 (6.3)
		3,001–7,345	68 (28.7)
		7,346–20,000	129 (54.4)
		≥20,001	25 (10.5)
	Marital status	Married	226 (95.4)
		Others (divorced/widow)	11 (4.6)

	Outcomes	Category	M	SD	Cutoff point	N (%)
Preschoolers outcome variables	PA	LPA	2012.9	391.4		
		MPA	788.7	233.6		
		Total MVPA	788.7	233.6		
		Average MVPA per day	116.1	30.1	≥60 min	191 (97.4)
					<60 min	5 (2.6)
		VPA	0	0		
	Sedentary	Average LSB	22.6	1.6	≥60 min	0
					<60 min	196 (100)
		Daily ASB	322.5	60.3		
		Sedentary	5563.5	1147.5		
	Sleep	Sleep latency	30.6	17.9		
		Sleep efficiency	77.2	6.7		
		Sleep duration (minutes)	445.9	38.6	≥10 h	0
					<10 h	196 (100)
	ST (minutes)	ST (weekday)	60.9	53.5	≥60 min	126 (53.2)
					<60 min	111 (46.8)
		ST (weekend)	123.7	104.7	≥60 min	188 (79.3)
					<60 min	49 (20.7)
	Eating style	EF	3.3	0.8		84 (35.4)
		EOE	1.8	0.6		2 (0.8)
		SR	3.0	0.64		27 (11.4)
		SE	3.1	0.84		55 (23.2)
		DD	2.4	0.9		14 (5.9)
		FF	2.7	0.7		20 (8.4)
		EUE	2.8	0.8		30 (12.7)
		FR	2.5	0.6		5 (2.1)
	Sleep	Sleep problems	50.2	6.9	Yes (score ≥ 41)	216 (91.1)
					No	21 (8.9)
Parents outcome variables	Parent’s PA(MET)	LPA	900.7	1245.1		
		MPA	365.4	913.4		
		VPA	655.8	1524.5		
	Parenting feeding style	IF	2.4	0.6		7 (3.0)
		EF	2.2	0.6		2 (0.8)
		EnF	3.5	0.7		103 (43.5)
		CE	3.5	0.5		125 (52.7)
	Parenting self-efficacy	In PA	28.7	8.0		
		In eating	41.9	10.4		
		In sleeping	7.5	2.2		
		In ST	15.2	4.2		

### The moderating effects on the association of PA-related parenting practices with preschoolers’ PA and sedentary behaviors

The marital status of parents was found to moderate the association of parents’ PA with preschoolers’ TMVPA (*B* = 0.679, *p* = 0.004). Parents’ PA and preschoolers’ TMVPA was positively related when parents get married (effect = 0.828, *p* = 0.012, 95 CI: [0.184: 1.471]), whereas this relationship found negative in single-parent family (effect = −0.155, *p* = 0.033, 95 CI: [−0.297: −0.012]).

The association between PA-related PSE and preschoolers’ LPA was found to be moderated by preschoolers’ gender (*B* = 0.319, *p* = 0.031). The PA-related PSE was negatively related to LPA for the girls (effect = −0.352, *p* = 0.002, 95 CI: [−0.576: −0.129]), but no longer related for the boys (*p* = 0.723). The relationship between PA-related PSE and preschoolers’ ALSB was moderated by the number of children in the household. This relationship was only negatively significant for family with more than one kid (effect = −0.20, *p* = 0.021, 95 CI: [−0.368: −0.030]), but not for the single child family (*p* = 0.274).

Preschoolers’ age moderated the relationship between parent’s PA and preschoolers’ sedentary behaviors (*B* = 0.388, *p* = 0.0001). Parent’s PA demonstrated a negative association with preschoolers’ sedentary behaviors for children aged at three (effect = −0.573, *p* = 0.011, 95 CI: [−1.010: −0.135]) and 4 years (effect = −0.329, *p* = 0.010, 95 CI: [−0.580: −0.078]), but not for peers aged at 5 years (*p* = 0.098).

### The moderating effects on the relationship between parents’ and preschoolers’ BMI

Parents’ educational level (*B* = −0.089, *p* = 0.008), household income (B = −0.114, *p* = 0.017), number of children in the household (*B* = 0.298, *p* = 0.018), and preschoolers’ age (*B* = −0.242, *p* = 0.014) moderated the relationship between parents’ BMI and preschoolers’ BMI. Specifically, parents’ BMI demonstrated a positive relationship with preschoolers’ BMI when parents were education level of college or lower (effect = 0.256, *p* = 0.000, 95 CI: [0.150: 0.362]). However, under the condition of parents with educational level beyond bachelor degree, this relationship was no longer significant (*p* = 0.079—0.524). When the household income ranged from 3,000 (effect = 0.341, *p* = 0.000, 95 CI: [206: 0.475]) to 20,000 RMB (effect = 0.170, *p* = 0.029, 95 CI: [0.017: 0.324]), there was a positive relationship between parents’ BMI and preschoolers’ BMI. However, this relationship was not observed when the income was below 3,000 or above 20,000 RMB (*p* = 0.090—0.226). There was a positive association between parents’ BMI and preschoolers’ BMI when there was more than one child in the household (effect = 0.237, *p* = 0.000, 95 CI: [0.145: 0.328]), but this association was not present in households with only one child (*p* = 0.592). A significantly positive relationship between parents’ BMI and preschoolers’ BMI was found when the children were 4 years old (effect = 0.495, *p* = 0.000, 95 CI: [354: 636]), but the relationship was absent when they were three and 5 years old (*p* = 0.266–925).

### The moderating effects on the association between parenting feeding practices and preschoolers’ eating behaviors

Regarding parenting feeding practices, preschoolers’ age moderated the relationship between parenting feeding practices and emotional overeating (*B* = 0.187, *p* = 0.046, 95 CI: [0.003: 0.371]). However, when exploring the moderating effect of age on the relationship between each feeding behavior (i.e., instrumental feeding, emotional feeding, encouragement to eat, and control eating) and the child’s emotional overeating, preschoolers’ age did not have a moderating effect on any of the four feeding behaviors and the child’s emotional overeating (*p* = 0.084—0.265). Preschoolers’ gender moderated the relationship between parenting feeding practices and preschoolers’ desire to drink (*B* = −0.438, *p* = 0.022). But no moderating effects of gender in relation to the each feeding practices and desire to drink was observed (*p* = 0.274—0.495). The relationship between parenting feeding practices and preschoolers’ food fussiness was found to be moderated by household income (*B* = 0.183, *p* = 0.034) and parents’ marital status (*B* = 0.804, *p* = 0.04). Neither household income (*p* = 0.295—0.876) nor parents’ marital status (*p* = 0.269—0.851) moderated the relationship between each feeding practices and preschoolers’ food fussiness.

Preschoolers’ age moderated the association between eating-related PSE and preschoolers’ slow eating (*B* = −0.150, *p* = 0.024) and preschoolers’ food fussiness (*B* = −0.120, *p* = 0.020). Specifically, PSE in eating is only negatively related with the preschoolers’ slow eating (effect = −0.250, *p* = 0.0006, 95 CI: [−0.039: −0.011]) and food fussiness (effect = −0.170, *p* = 0.003, 95 CI: [−0.029: −0.006]) in children aged at 5, but not in 3-year-old (*p* = 0.354—0.472) and 4-year-old children (*p* = 0.053—0.082). The relationship between eating-related PSE and preschoolers’ food fussiness was also moderated by parents’ marital status (*B* = −0.072, *p* = 0.020). Compared to children raised in single parent family, married parents with eating-related PSE were more likely to decrease the food fussiness in preschoolers (−0.104 vs. −0.083, *p* = 0.007—0.014).

### The moderating effects on the association of sleep-related PSE with preschoolers’ sleep duration

The relationship between sleep-related PSE and preschoolers’ sleep duration was moderated by the number of children in the household (*B* = −0.343, *p* = 0.034). Sleep-related PSE was positively related with preschoolers’ sleep duration in only child family (effect = 0.247, *p* = 0.046, 95 CI: [0.005: 498]), but not in family with more than one child (*p* = 0.262).

## Discussion

This study aims to investigate the heterogeneity in the association between parenting practices and preschoolers’ EBRBs based on preschoolers’ age and gender as well as parents’ marital status, income, number of children in the household, and educational level. Understanding how these demographic characteristics affected the relationship between parenting practices and preschoolers’ EBRBs in China is important for developing tailored family-based programs for future studies. Collectively, age of preschooler moderated the several associations of parenting practices and EBRBs of preschoolers (i.e., parents’ and preschoolers’ BMI, parents’ PA and preschoolers’ sedentary behaviors, eating-related PSE correlates of preschoolers’ slow eating and food fussiness). Gender of preschooler moderated the relationship between PA-related PSE and preschoolers’ LPA. Additionally, marital status of parent influenced the association of parents’ PA with preschoolers’ MPA/MVPA and correlation of eating-related PSE with preschoolers’ food fussiness. Income moderated the relationship between parents’ and preschoolers’ BMI. Number of children in the household moderated the following relationships (parents’ and preschoolers’ BMI, PA-related PSE and preschoolers’ ALSB, and sleep-related PSE and preschoolers’ sleep duration). Educational level moderated the relationship between parents’ and preschoolers’ BMI.

Age of preschoolers showed different moderating trend on the relationship between parents’ PA and preschoolers’ sedentary behaviors as well as the association of eating-related PSE with preschoolers’ eating behaviors (i.e., slow eating and food fussiness). Specifically, the influence of parents’ PA on preschoolers’ decreased sedentary behaviors was more pronounced in 3-year-old children and in 4-year-old children, but was absent in 5-year-old children, whereas the associations of eating-related PSE with preschoolers’ slow eating and food fussiness were only significant in 5-year-old children but not in children aged at 3 and 4 years. The former moderation may reflect that younger preschoolers (3–4 years) may be more dependent on and responsive to their parents’ modeling and encouragement of PA, leading to reduced sedentary time (40). As preschoolers grow, they undergo significant physical, cognitive, and social–emotional development, older preschoolers (5 years) may become more independent and influenced by factors outside the immediate family, such as peer relationships and school environments, attenuating the direct impact of parents’ PA on their sedentary behaviors (41, 42). More importantly, Chinese parents have long placed great importance on education, believing that earlier and high academic achievements can lead to more successful career future and economic success (43–47). Chinese children are facing greater academic burden and higher expectations from their parents, which has led to the phenomenon of “educational involution” (*neijuan* in Chinese) (48–50). Parents were more likely to force their children as young as 5 years old (also known as transition stage from preschool to primary school in China) to participate in numerous self-care and academic-related activities to avoid falling behind (43). Another unique feature of China education system is that the enrollment rates vary significantly across provinces in China due to differences in socioeconomic status, the unequal geographic distribution of high-quality educational resources, and varying admission score requirements (51). Students from Guiyang (the undeveloped city in China where the data was collected) could be motivated to spend huge amounts of time and energy to pursue high scores in Zhongkao (High School Entrance Examination) and Gaokao (National College Entrance Examination) than their counterparts in advantaged regions, in hopes of gaining admission to top universities (52). This overemphasis on academic achievements potentially neglect the importance of less sedentary behaviors. In addition, any activities such as PA that may compromise examination results may be gradually eliminated from a very young age (53, 54). These potential explanations do not appear to be the case in the latter moderation. This may be due to food neophobia, a natural developmental stage characterized by the tendency of young children to reject or be reluctant to try new or unfamiliar foods (55). This picky eating behavior can be observed as early as the first year of life, but typically intensifies around 2 years old. This heightened aversion to novel foods is driven by innate factors during this early childhood. After reaching this peak, the level of nutritional neophobia gradually declines as children transition into adolescence and adulthood and shows a slight increase in old age (55). Although neophobia is genetically determined, it can be reversed by the environment in which the child is raised (e.g., family, peers, and parents) (56). The developmental progression shows a shift from innate and biologically driven food preferences in early childhood to a greater influence of learned and social factors on food choices as children mature (57–59). Therefore, 3- and 4-year-old children were more likely to be influenced by their innate food preferences and exhibit picky eating behaviors compared to 5-year-old children. As children grow older, their food preferences become increasingly shaped by environmental and social factors, such as parental modeling, exposure to new foods, and education about nutrition (60–62).

The relationship between PA-related PSE and preschoolers’ PA has been demonstrated in the previous studies (63, 64), but this relationship differed for the gender of preschoolers in the current study. PA-related PSE was negatively associated with girls’ PA but not related to boys’ PA. This may be because that females are viewed as inherently inferior and subordinate to males in the traditional Chinese Confucius ideology (65). From a young age, Chinese girls and boys are segregated and receive different treatment to prepare for their respective future roles (66). Girls may be more susceptible to significant societal and cultural pressure to conform to feminine norms (67). Girls are expected to be gentle, kind, and sensitive, PA are deemed inappropriate for them (17). Parents may hold passive attitudes and expectations when it comes to encouraging PA for their daughter, this concept could diminish the positive impact of PSE (17).

The moderating effects of parents’ marital status indicated that married status produced desired outcomes between parenting practices and preschoolers’ EBRBs while divorced status found unfavorable results. Specifically, the relationship between parents’ and preschoolers’ PA was positive when the parents were married but negative when they were divorced. Besides, married parents with PSE in eating were more likely to alleviate preschoolers’ food fussiness compared to divorced parents (−0.104 vs. − 0.083, *p* < 0.05). These findings accord with earlier observations. For instance, previous studies exploring the relationship between family circumstances and children’s PA demonstrated that children raised in dual-parent families were more likely to engage in PA while children with single-parent household were less physically active (68–70). A large-scale cross-sectional study of Piloquet et al. (71), who investigated the food fussiness correlates of family environmental factors, indicated that food fussiness measured by CEBQ (which is the same as the current study measurement used to assess eating behaviors) was higher in children who lived with only one of their parents. These findings could be attributed to crucial role family plays in preschoolers’ behavior development. Based on the Family Systems Theory proposed by Bowen (72), which is a framework for understanding human behavior that views the family as an emotional unit and employs systems thinking to describe the complex interactions within that unit. Family members are intensely connected emotionally, often seeking each other’s attention, approval, and support while responding to one another’s needs, expectations, and distress (72). This interconnectedness creates interdependence among family members, meaning that a change in one person’s behavior is likely to prompt reciprocal changes in others (72). Within this social system, families possess unique characteristics, rules, roles, communication patterns, and power structures that extend beyond individual members (73). Families also consist of various subsystems, such as parent–child, sibling, marital, and co-parenting subsystems. Each family member influences each other directly and indirectly, with one member potentially affecting the dynamics between others (73). Interactions within families are viewed as transactional, meaning that parent–child interactions are bidirectional rather than one-sided, and causality operates in a circular manner rather than a linear one (73). Importantly, research has shown that parents’ negative emotions stemming from marital issues can “spill over” into their relationships with their children, potentially leading to the child’s maladjustment (74). Zheng et al. (75) highlighted the positive association between intact family and children’s PA was based on the hypothesis that children living with dual-parent family have more access to the beneficial entities of parental influence (e.g., role modeling and support of PA) compared to children living with single parent. Moreover, in married families, parents typically have a more cohesive emotional bond, which can foster supportive environments for children. This emotional support may encourage both parents and preschoolers to engage in PA together, leading to a positive relationship between their levels of PA (68). In contrast, divorced parents might experience emotional distance and conflict, which can reduce the quality of parent–child interactions (68). This lack of emotional support can negatively affect the motivation and opportunities for PA, resulting in a weaker or negative relationship between parents’ and preschoolers’ PA. Hasenboehler et al. (76), who evaluated the association between family structure and children’s eating behaviors, indicated that family cohesion (i.e., emotional closeness with children) was related to decreased restrained eating of children (i.e., attempts to refrain from eating). This may be because in a married context, parents were likely to work together, reinforcing each other’s strategies and decisions regarding their child’s eating habits. This collaboration may lead to consistent approaches that effectively reduce food fussiness. On the other hand, divorced parents may have differing approaches or conflicting strategies due to their separate households, which can confuse the child and exacerbate food-related issues (73, 76).

The mechanism linking the sleep-related PSE and preschoolers’ sleep duration varied by the number of children in the household, sleep-related PSE demonstrated a positive relationship with preschoolers’ sleep duration in single-child family, but this relationship was not significant in family with more than one child. This phenomenon may be because in single-child families, parents can devote more attention and involvement in their child’s sleep routines and more easily implement sleep-related strategies such as establishing and maintaining more control over the child’s sleep environment (77). Whereas, with multiple children, coordinating sleep schedules and routines for multiple children can be more challenging because parents may need to divide their time and resources (78), potentially reducing the intensity of their involvement in each child’s sleep (79). This finding also accords with previous study, which indicated that the sleep patterns and routines of the parent or sibling that the child shares a room with can have a significant influence on the child’s own sleep duration and quality. The child may feel compelled to synchronize their sleep–wake cycles with those of the person they are sharing a room with, rather than being able to follow their own optimal sleep schedule. These sibling-related sleep disturbances may diminish the positive impact of sleep-related PSE (80).

By examining the moderating effects of demographic characteristics, the study sheds light on the importance of considering the broader social and family context when examining the parenting-EBRB relationship. However, this study also contains several limitations. Data related to parents, preschoolers’ eating behaviors and preschoolers’ sleep problems and quality were subjectively assessed using parent-reported questionnaires, which may have led to report bias and inaccuracy. In the current study, the unequal distribution of preschoolers’ age and gender as well as parents’ demographic characteristics may mask or exaggerate the moderating effects. Therefore, further research with a more balanced distribution is necessary. Questionnaire items assessing sleep-related PSE were limited, which may have compromised the sensitivity of the measures. The number of moderators tested were limited by the data that were collected in the baseline of main intervention study. Finally, caution is warranted when generalizing our results to the broader population of young children raised in developed regions in China.

## Conclusion

The current study extends the existing literature by demonstrating the moderating effects of demographic background on the relationship between parenting practices and preschoolers’ EBRBs. Married parents exhibited the desired outcomes in the link between parenting practices and preschoolers’ EBRBs. Conversely, divorced or separated parents showed unfavorable results in this relationship. The relationship between parents’ and preschoolers’ BMI varied depending on parents’ educational level, household income, preschoolers’ age, and number of child in the household. The age of the preschoolers moderated the relationships between parents’ PA and preschoolers’ sedentary behaviors as well as the association of eating-related PSE with preschoolers’ slow eating and food fussiness. The mechanism linking PSE and preschoolers’ PA, sedentary behaviors and sleep duration were influenced by preschoolers’ gender and number of children in the household. These findings represent an important advancement in understanding the contextual influences on the parenting practices-children’s EBRBs relationship, which suggests that interventions should be multifaceted, considering factors such as age and gender sensitivity, family structure, economic factors, parental status, and educational attainment. By tailoring health promotion strategies to these moderating factors, practitioners and policymakers can better support families in fostering healthier behaviors in preschoolers.

## Data Availability

The raw data supporting the conclusions of this article will be made available by the authors, without undue reservation.
